# Bacteriological Diagnosis

**Published:** 1892-06

**Authors:** 


BACTERIOLOGICAL DIAGNOSIS. Tabular aids for use in practical work, by James Eisenberg, Ph. D., M. D., Vienna. Translated and augmented, with the permission of the author, from the second German edition by Norval H. Pierce. Philadelphia (1231 Filbert Street), and London. The F. A. Davis Co., Publishers. 1891. Price, $1.50, net.

This volume gives in tabular form all the information obtainable concerning the different species of bacteria with a view to the recognition of any species under the microscope.

All bacteria are divided according to this book into I, Non-pathogenetic Bacteria; II, Pathogenetic Bacteria; III, Fungi. The Non-pathogenetic are sub-divided into A, liquefying gelatine, and B, not liquefying gelatine. The Pathogenetic are divided into A, cultivated outside the animal body; B, not cultivated outside the animal body.

Each individual variety occupies a page by itself, and the reactions under the microscope, and conditions of growth by which it may be recognized are concisely given. The work is particularly useful for the practical microscopist.

